# Long-term changes in the ghrelin-CB1R axis associated with the maintenance of lower body weight after sleeve gastrectomy

**DOI:** 10.1038/nutd.2014.24

**Published:** 2014-07-14

**Authors:** C Fedonidis, N Alexakis, X Koliou, O Asimaki, E Tsirimonaki, D Mangoura

**Affiliations:** 1 Center for Neurosciences, Biomedical Research Foundation of the Academy of Athens, Athens, Greece; 2 Department of Surgery, Medical School, University of Athens, Athens, Greece

## Abstract

**Objectives:**

In the hypothalamus, the molecular actions of receptors for growth hormone secretagogue (ghrelin) receptor-GHSR, leptin receptor-b (LEPRb), Melanocortin-4 receptor (MC4R) and Cannabinoid-1 receptor (CB1R) regulate energy homeostasis and body weight. We hypothesized that the acute loss of stomach tissue upon sleeve gastrectomy (SG), performed to treat obesity, imposes modulations on the expression of these receptors in the brain to sustain weight loss.

**Methods:**

Rats, induced to obesity with high-fat diet were randomized to SG- or sham-operation groups and killed at 30 or 90 days post surgery, when the expression of *Ghrl*, *Mboat4* and *Cnr1* in the stomach, and *Ghsr*, *Leprb*, *Mc4r* and *Cnr1* in distinct brain areas was assessed by reverse transcription-PCR and western blotting.

**Results:**

SG acutely reduced body weight and fat mass and suppressed the remnant stomach mRNA levels of preproghrelin and ghrelin *O*-acyltransferase, which correlated well with long-term decreases in CB1R mRNA. In the hypothalamus, increases in GHSR and decreases in CB1R and LEPRb by 30 days were followed by further downregulation of CB1R and an increase in MC4R by 90 days.

**Conclusions:**

Post SG, acyl-ghrelin initiates a temporal hierarchy of molecular events in the gut-brain axis that may both explain the sustained lower body weight and suggest intervention into the cannabinoid pathways for additional therapeutic benefits.

## Introduction

An established surgical procedure to effectively manage severe obesity is sleeve gastrectomy (SG),^1, 2^ whereby resection of the greater curvature of the stomach reduces gastric volume by almost 80%. Following SG, acute loss of excess weight up to 80% by the first year^2^ stabilizes at ∼50% by 6–8 years.^1, 3^ Although diet regimens fail to maintain weight loss through the regulation of hormonal levels,^4^ SG has long-lasting effects on loss of excess weight, as a unique hormonal balance is established between the plasma levels of ghrelin, the hormone that promotes food intake, and those of leptin that inhibits it.^5, 6, 7, 8^ This balance leads to metabolic improvements^9^ and changes in food preferences, which shift from high fat foods to lower caloric foods.^10^ The volume restrictive features of this procedure alone do not fully explain these effects,^8^ rather point out a centrally regulated interplay between the plasma levels of hormones that regulate energy homeostasis and their receptors in the brain.

The gut-brain axis is controlled by the hypothalamus to maintain energy homeostasis through signaling of the membrane receptors growth hormone secretagogue receptor (GHSR), Leptin receptor-b (LEPRb), Melanocortin-4 receptor (MC4R), and Cannabinoid-1 receptor (CB1R).^11, 12, 13^ Ghrelin, mainly produced by the A-like cells of the stomach, increases both food intake and fat storage. Preproghrelin, the protein product of the *Ghrl* gene, is acylated by ghrelin-*O*-acyltransferase (GOAT), the product of the *Mboat4* gene,^14^ to produce the active acyl-ghrelin that binds GHSR and hence activates the orexigenic pathway in the arcuate nucleus of the hypothalamus.

Counteracting ghrelin, leptin, produced by the adipose tissue at proportional levels to the stored fat, acts through hypothalamic LEPRbs to both inhibit food intake and promote lipid mobilization. Leptin stimulates the anorexigenic pathway mainly through the production of α-MSH,^12^ the specific agonist of MC4R that inhibits feeding behavior.^15^ Feeding inhibition may be, however, ablated by the endogenous MC4R antagonist agouti-related protein; production and secretion of this appetite-stimulating neuropeptide is induced in response to ghrelin and diminished in response to leptin.^12^ The importance of MC4R was further documented by the development of obesity in humans carrying *MC4R* gene mutations, whereas in MC4R-knockout mice, hyperphagia and obesity is reversed when MC4R is reexpressed in the hypothalamus.^16^

Equally important for appetite and metabolism regulation is the endocannabinoid (EC) system, consisting of the cannabinoid ligands (ECs) and their widely expressed receptors (CB1Rs). The availability of CB1R ligands may positively regulate its expression, as also shown for GHSR, LEPRb and MC4R.^17, 18, 19, 20, 21^ Leptin reduces the hypothalamic production of ECs,^22^ whereas ghrelin elevates it to promote appetite; the latter—given the actions of CB1R on peripheral organs—in conjunction with unhealthy dieting practices may lead to increased risk for adiposity.^13, 23, 24^ Thus, CB1R, a pleiotropic receptor that controls central neural functions,^25^ may mediate the effects of both ghrelin and leptin.

Therefore, we undertook these studies to investigate transcriptional and translational regulation of CB1R, MC4R, LEPRb and GHSR receptors following SG-removal of ghrelin-producing stomach tissue, which leads to sustained weight loss in the long term. More specifically, we assessed the temporal changes in receptors' mRNA and protein expression levels in distinct areas of the central nervous system in a rat model of SG at 30 and 90 days post SG, which correspond to 3 and 8 years in humans and thus cover the reported periods of weight regain after SG.^3^ Elucidating the molecular mechanisms that control the maintenance of weight loss post SG may lead to better intervention into obesity.

## Materials and methods

### Animals

According to protocols approved by the Hellenic Regional Animals Authority (#K1492/2006), 26 male 10-week-old Wistar rats, individually caged under pathogen-free stable conditions (20–24 °C, 50–60% relative humidity, 12 h-light/dark cycle), followed a chow diet (Harlan Teklad-F6–8664; 3.1 kcal g^−1^) referred to as normal diet. To establish obesity, animals were fed high-fat diet (HFD) (Harlan Teklad-TD-88137; 4.5 kcal g^−1^) for 12 weeks and reached a mean body and fat mass weight of 530±16 and 84.65±8.93 g, respectively, as compared with an average of 400±11and 26±1.5 g in animals that were kept in normal diet. Then some animals were killed to serve as obese control, whereas the rest were randomly assigned into two groups, subjected to either SG or sham operation, and killed 30 or 90 days later. In rats, acute weight and food intake reductions occur during the first 2–3 weeks post SG,^10, 26^ thus, the time points of 30 or 90 days were considered as suitable to study phenotypic changes. Post surgery, all animals were fed normal diet *ad libitum* as for all experimental conditions.

### Surgical procedures

Rats were deprived of food for 16–18 h prior to operation. Anesthesia was established with ketamine and xylazine (200 mg: 5 mg, 0.8 ml kg^−1^, intraperitoneally). SG (removal of 70–80% of the stomach) was performed through a mid-line incision, using a laparoscopic linear stapler (Ethicon Endosurgery, Cincinnati, OH, USA) equipped with the ETS45 2.5-mm vascular cartridge. Sham operation refers to stomach exposure out of the peritoneal cavity and then closure of the abdomen. During the first three post operative days, animals received buprenorphine hydrochloride 0.05 mg kg^−1^ twice a day and were hydrated with normal saline solution (20 ml) injections, whereas a liquid supplement was provided after 24 h for 6 days.

### Tissue harvesting and processing

Tissues were collected from five study groups: obese (time of surgery, *n*=4), and sham (*n*=10) or SG (*n*=12) at 30 or 90 days post surgery. Brain areas (hypothalamus, hippocampus, cerebral cortex, cerebellum, amygdale, and olfactory bulbs) were bilaterally dissected out,^27^ randomly divided for either mRNA or protein analysis and placed in Trizol or RIPA buffer (containing protease inhibitors), respectively. Gastric tissue was collected from the upper part of the stomach (close to gastroesophageal junction). Subcutaneous abdominal and mesenteric, retroperitoneal and epididymal adipose tissue was dissected out and weighted to depict changes in fat mass.

Semi-quantitative reverse transcription-PCR was performed as described.^28^ Briefly, total RNA isolated from brain areas or gastric tissue using the Trizol reagent (Ambion, Grand Island, NY, USA) was reverse transcribed (1 μg per reaction) with SuperScript II reverse transcriptase (Invitrogen, Grand Island, NY, USA), and 1 μl of the formed cDNA was amplified with Taq DNA Polymerase (Hytest, Turku, Finland), in the presence of gene-specific primers (Table 1). Amplification conditions were an initial denaturation step of 4 min at 94 °C followed by 32–37 cycles (linear region) at 94 °C for 30 s, at 50–58 °C (depending on annealing temperature of each primer set, Table 1) for 30 s, at 72 °C for 30 s, followed by 10 min at 72 °C. PCR products, separated in appropriate for each product percentage agarose gels, were stained with ethidium bromide and visualized under UV light using the Dolphin-Doc Pro system (Wealtec, Taipei, Taiwan). For quantification, band intensities were assessed using the ImageJ software (http://imagej.nih.gov/ij/) and normalized in reference to *Gapdh* gene expression, which was always amplified for each cDNA preparation in its linear region of 23 cycles. Pilot experiments established the minimum amplification cycles that reflected the linear region of the band intensities of the studied genes and *Gapdh* mRNA levels.

SDS-PAGE, western blotting (WB), and enhanced chemiluminescence were performed as described.^25, 27^ Primary antibodies for GHSR 1:5000, LEPRs 1:1000, MC4R 1:5000 and preproghrelin 1:1000 were purchased from Santa Cruz (Dallas, TX, USA) and β-actin 1:2000 from Sigma (St Louis, MO, USA). Pilot experiments established that chemiluminescence detection was linear (for 25–60 μg of protein) at 10 min of exposure for all antibodies, except for actin that was linear at 2 min. Exposed films were scanned for densitometric analysis, and after the protein detection levels were normalized to actin detected levels.

### Statistical analysis

We used multivariate analysis of variance tests in order to detect any statistically significant relationships between changes in the type of operation and/or the time point of tissue harvesting. Levene's test of equality of error variances was used to assess the null hypothesis that the populations from which the samples were drawn have comparable variances. Multiple pair wise comparisons using Tukey's HSD *post hoc* tests were also performed in order to determine statistically significant changes in the measured variables between groups. Statistical significance level (*P*) was set at 0.05. The IBM SPSS Statistics software package v22.0 (IBM Corp., Armonk, NY, USA) was used to perform the statistical analysis.

## Results

### SG reduced body weight and adipose tissue in a rat obesity model

**Figure 1:**
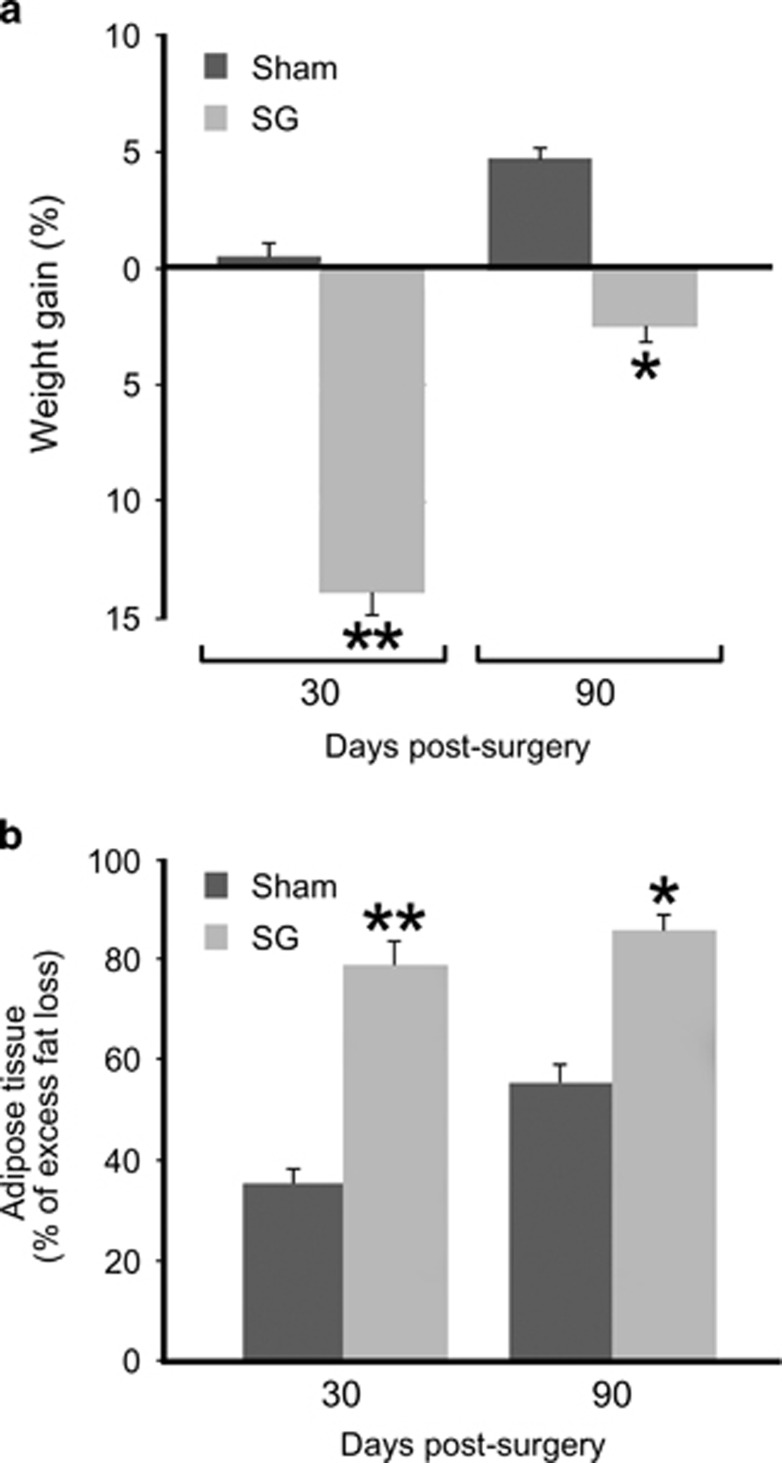
Body weight and fat mass reductions following sleeve gastrectomy (SG). (**a**) The plot shows gains in body weight at 30 or 90 days after the operations (sham or SG), and the concomitant switch from HFD to normal diet. The weight differences are expressed as a percentage of the change from the respective obese weight, at the time of surgery for each animal group, (*x* axis). (**b**) The loss in adipose tissue of the killed animals is expressed as a percentage of the excess fat mass gained during the period of HFD for each group: SG animals lost >80% of the fat mass they gained during HFD, whereas in the sham-operation group the loss of excess fat mass was only 50%. *P< 0.05 and **P< 0.01, asterisks indicate significant differences of body weight between sham- and SG-operated animals in (**a**) and adipose tissue in (**b**) at 30 & 90 days post surgery. *n*=4–6 animals per group.

Animals who underwent SG had lost 14% of their weight at the time of the surgery, and this loss progressively led to a level of 2.5% below their own obese weight by 90 days post surgery. The weight of the sham-operated animals, however, recovered and increased by 90 days, relative to the weight at the time of the surgery (Figure 1a). The significant differences in body weight between SG- and sham-operated animals, documented at 30 (*P*<0.01) or 90 days (*P*<0.05) post surgeries (Figure 1a), were most likely due to the greater loss of the excess fat mass seen in SG animals relative to their sham counterparts (*P*<0.01 and *P*<0.05; Figure 1b).

### SG suppressed the gastric expression of key molecules involved in acyl-ghrelin homeostasis

**Figure 2:**
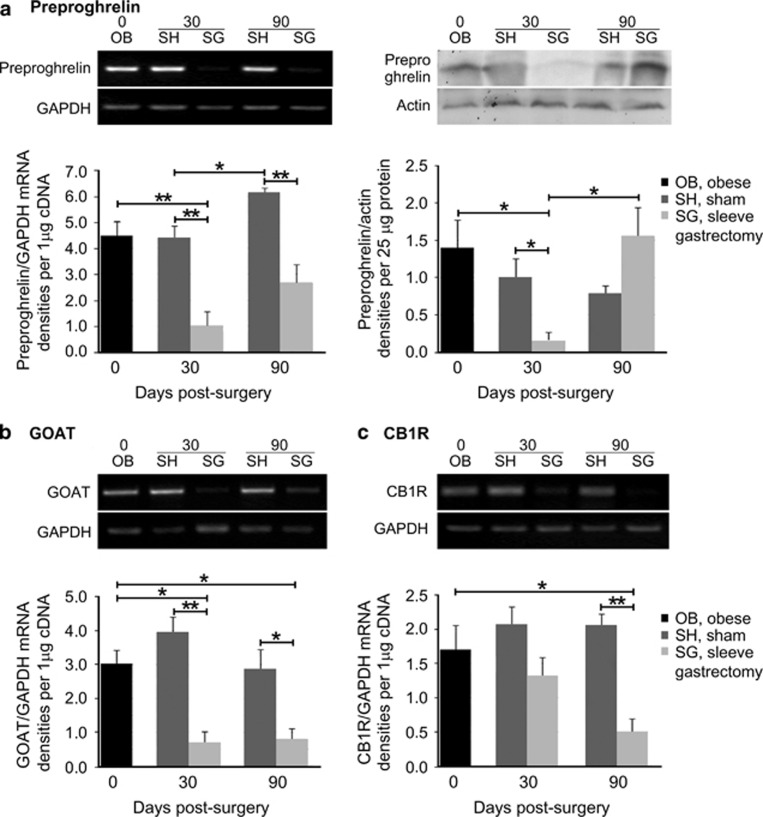
Regulation of the gastric ghrelin system by SG. (**a**) Sustained reduction of preproghrelin mRNA post SG (left panel); detection of the non secreted preproghrelin peptide levels exhibited a decrease only in the short term, whereas peptide levels recovered almost fully in the long term (right panel). mRNA levels analysis of (**b**) GOAT and (**c**) CB1R showed maintenance of low *Mboat4* expression levels, in contrast to the decrease in CB1R mRNA expression, which reached significance at 90 days after SG as compared with sham-operated animals. Representative images of reverse transcription (RT) PCR and WB are illustrated on top of the respective graphs. The expression levels of the housekeeping markers *GAPDH* and actin were used to normalize the genes of interest expression levels for RT-PCR and WB, respectively. *n*=4–6 animals per group; *P< 0.05 and **P< 0.01.

To investigate whether changes in the ghrelin-producing system in the remnant stomach could be involved in the maintenance of lower plasma levels of acyl-ghrelin following SG, we examined the expression of the preproghrelin gene. We observed a significant reduction of preproghrelin at both the mRNA (Figure 2a, left) and the protein level (Figure 2a, right) at 30 days post SG, as compared with post sham surgery (*P*<0.01 and *P*<0.05, respectively). By 90 days post SG, preproghrelin mRNA levels had partially recovered, but still were significantly lower than those in post sham (*P*<0.01); preproghrelin protein levels of SG rats did not, however, significantly differ from that of sham-operated animals (Figure 2a).

The levels of *Mboat4* were significantly suppressed in rats subjected to SG relative to those subjected to sham surgery, at both 30 or 90 days post surgery (*P*<0.01 and *P*<0.05, respectively; Figure 2b). Therefore, suppression of *Mboat4* expression may be part of the same transcriptional program that reduces the production of acyl-ghrelin post SG. This downregulation was, however, more profound than the one observed with ghrelin, indicating even lesser acylation of the available ghrelin molecules.

Reduced plasma ghrelin levels are linked to blockade of CB1R signaling,^29^ thus, we examined the expression of CB1R mRNA in the stomach. We found a trend towards lower CB1R mRNA levels by 30 days, which reached significance by 90 days post SG (*P*<0.01; Figure 2c). This decrease in CB1R expression correlated well with the increase at preproghrelin protein levels of SG animals at 90 days, compared with 30 days (Figure 2a). Thus, in addition to lower ghrelin acylation, downregulation of CB1R expression may also contribute to decreases in the availability of acyl-ghrelin from the remaining stomach in the periphery.

### Differential control of energy homeostasis-related receptors at the hypothalamus in sleeve gastrectomized rats

**Figure 3:**
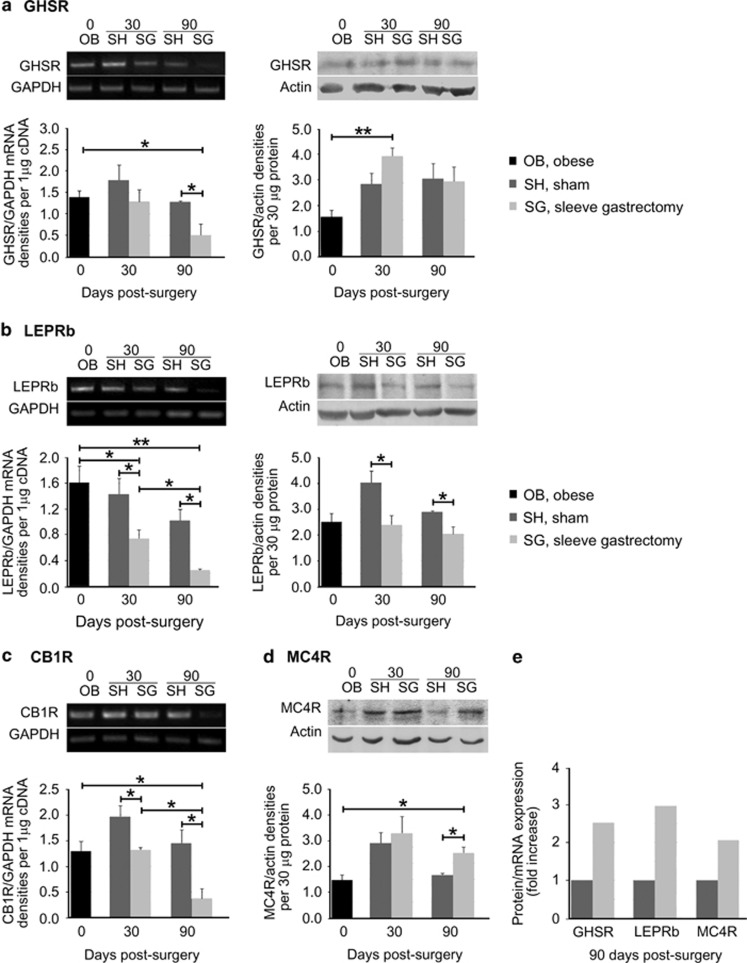
SG alters the mRNA and protein levels of receptors in the hypothalamus. (**a**) Detection of *Ghsr* expression levels by semiquantitative RT-PCR revealed a significant decrease in the mRNA levels of SG animals at 90 days post surgery, as compared with sham or obese animals, whereas GHSR protein levels, detected by WB, increased significantly post SG relative to obese animals. Following SG, (**b**) the expression of *Leprb* at both the mRNA and protein levels was constantly significantly lower from that of their sham counterparts; this effect is also observed for (**c**) CB1 receptor message levels. (**d**) MC4R protein levels in the hypothalamus showed a significant upregulation by 90 days after SG, as compared with sham surgery. (**e**) Calculation of the protein over mRNA ratio provided evidence for the expression levels of each receptor relative to the mRNA. The protein/mRNA ratio of the sham group for each receptor was considered as 100%. By 90 days post SG GHSR exhibited a 2.5-fold, LEPRb a threefold and MC4R a twofold increase of their expression levels. Representative images of RT-PCR and WB are illustrated on top of the respective graphs. The expression levels of the housekeeping markers *GAPDH* and actin were used to normalize the genes of interest expression levels for RT-PCR and WB, respectively. *n*=4–6 animals per group; *P< 0.05 and **P< 0.01.

In the hypothalamus of SG animals, we found a significant downregulation of *Ghsr* transcription, as compared with the sham-operated or the obese animals, in the long term (90 days; *P*<0.05; Figure 3a, left). We observed an increase in GHSR protein levels 30 days post SG, relative to those of the obese animals (*P*<0.01). By 90 days post SG, however, this upregulation declined and reached the same levels as in the sham group (Figure 3a, right).

When we examined the message levels of LEPRb, we found that at 30 or 90 days postsurgeries, the expression of this mRNA was significantly lower in the SG group as opposed to the sham group (*P*<0.05; Figure 3b, left). A similar pattern was observed for the protein levels of the receptor, which declined accordingly (*P*<0.05; Figure 3b, right).

We documented a persistent downregulation of CB1R message levels in SG, as opposed to sham animals, throughout the study period (*P*<0.05; Figure 3c). In the protein levels of the MC4R, we observed a paradoxical regulation. More specifically, whereas mRNA levels did not change (data not shown), an initial trend towards upregulation of MC4R protein expression became significant by 90 days (*P*<0.05; Figure 3d).

To better portray these differential regulations in the mRNA or protein levels of the energy-related receptors in the hypothalamus, we plotted mRNA vs protein (Figure 3e). Apparently, no significant differences were observed for the ratio of protein/mRNA levels of the GHSR, LEPRb and MC4R at 30 days post SG in comparison with sham. By 90 days post operations, however, in SG animals, depletion of ghrelin led to increased abundance of GHSR, as the protein/mRNA ratio post SG was 2.5-fold higher than that seen after sham surgery. Likewise, LEPRb and MC4R exhibited increased ratios of threefold and twofold, respectively (Figure 3e). Collectively, these data may indicate that mechanisms that regulate protein availability, such as the rate of ligand-dependent endocytosis and/or degradation, may be altered in the long term after SG to increase protein abundance.

### The expression of appetite-related receptors was influenced by SG at extra-hypothalamic areas

We investigated the expression of energy-related receptors in extra-hypothalamic areas post SG and detected changes in the hippocampus and the cerebellum, but not in the amygdala, the brain cortex and the olfactory bulbs.

**Figure 4:**
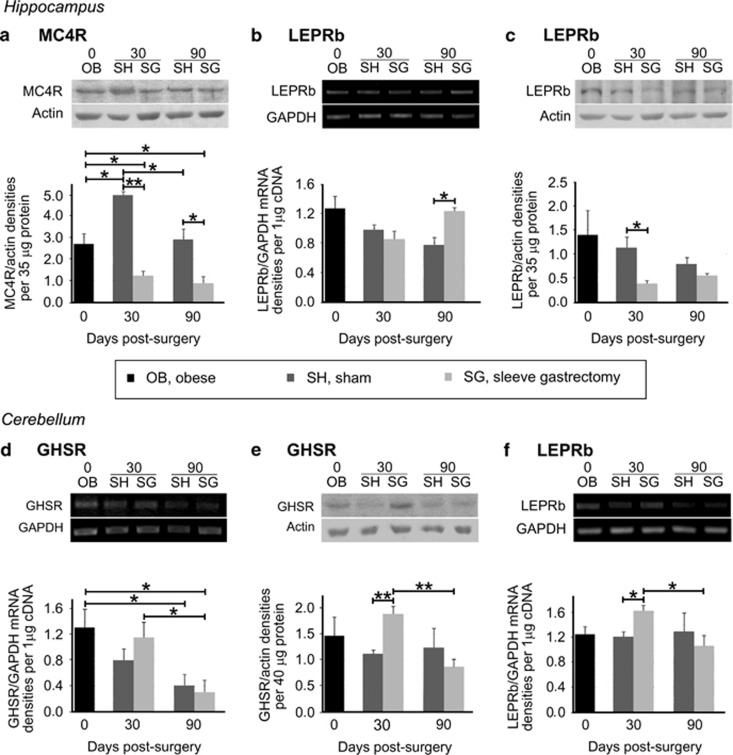
Energy homeostasis-related receptors exhibit differential expression levels in extra-hypothalamic areas after SG. In the hippocampus of SG animals a decrease in (**a**) MC4R protein levels were documented throughout the study period. In the same brain area, (**b**) LEPRb mRNA was solely increased in SG relative to sham-operated animals at 90 days post interventions, whereas (**c**) LEPRb protein levels reduced at 30 days post SG. In the cerebellum, a trend towards increased (**d**) GHSR message at 30 days post SG resulted in a significant increase at (**e**) protein levels of GHSR. In the long term, GHSR mRNA levels decreased in the SG group as compared with the levels of SG animals 60 days earlier. This decrease in message produced a decrease in GHSR protein levels between the SG animal groups. (**f**) The mRNA of LEPRb in the cerebellum increased only in the SG group, as opposed to sham, at 30 days post surgeries. Representative images of RT-PCR and WB are illustrated on top of the respective graphs. The expression levels of the housekeeping markers *GAPDH* and actin were used to normalize the genes of interest expression levels for RT-PCR and WB, respectively. *n*=4–6 animals per group; *P< 0.05 and **P< 0.01.

In the hippocampus, a significant effect of SG at the levels of MC4R was documented. Although, the mRNA levels were not significantly altered (data not shown), a prominent reduction in MC4R protein levels was sustained for up to 90 days post SG relative to sham animals (*P*<0.01 and *P*<0.05; Figure 4a). Moreover, the message levels of LEPRb in SG animals, similar to those in sham animals by 30 days following surgery, were apparently increased by 90 days (*P*<0.05; Figure 4b). On the contrary, the protein levels of LEPRb, initially reduced by SG after 30 days in comparison with sham surgery (*P*<0.05), but were not significantly affected in the long term (Figure 4c).

In the cerebellum, comparison of the GHSR message levels between SG animals at 30 and 90 days post surgery revealed a significant decrease in the SG group (*P*<0.05), which correlated with a drastic decrease in GHSR protein abundance (*P*<0.01; Figures 4d and e). Moreover, at 30 days following surgery, an increase of LEPRb mRNA was detected in the cerebellum of SG animals as compared with sham (*P*<0.05; Figure 4f). Although this difference dissipated, a significant time-depended decrease was documented in the SG group between the studied time points (*P*<0.05; Figure 4f).

## Discussion

The molecular mechanisms that act in the gut-brain axis to sustain the weight loss in the long term following SG remain unclear. To address these questions we have studied the expression of receptors that control energy homeostasis in rats who have undergone SG, a model that simulates the human post SG condition. Indeed, the weight of the SG animals in our study, kept in chow diet post surgery, was 15% less than their obese weight, even at 30 days post SG, mostly due to an ∼80% loss of their excess fat mass. In the long term, no further significant weight/fat loss or regain was observed in SG animals, in line with the observations that the greatest losses usually occur acutely by 2 weeks post SG.^10, 26^ These findings suggest that by 30 days post SG, a new lower body weight ‘set point' was established and maintained thereafter.

In addressing the mechanism of this sustained fat loss, we found overall that the acute SG-induced loss of ghrelin-producing tissue and the lower circulating acyl-ghrelin levels post SG^5, 6, 7^ initiated changes into receptors expression in a temporal fashion that hierarchically, from the stomach to the brain, modulated the orexigenic and anorexigenic pathways. Specifically, when we investigated the ghrelin-producing capacity of the endocrine cells in the remnant stomach, we found that expression of preproghrelin, the non-secreted form of ghrelin, was significantly decreased by 30 days post SG. Although mRNA levels remained significantly lower than their sham-operated counterparts, in line with previous observations,^30^ protein levels recovered after 90 days post SG, most likely due to lack of adequate acylation by GOAT and intracellular retention. Indeed, the significant reduction in GOAT expression was the most permanent effect post SG, hence the low plasma levels of acyl-ghrelin reported previously.^5^

**Figure 5:**
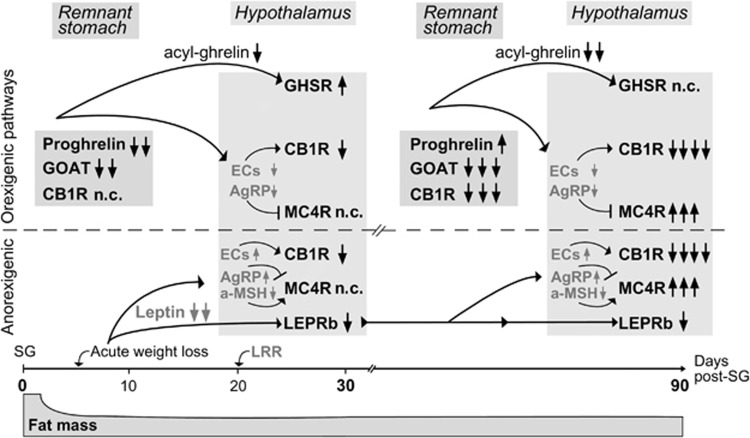
Proposed model of the gut-brain axis molecular mechanisms involved in the maintenance of lower body weight after SG. The acute loss of gastric tissue by SG decreases the expression of both ghrelin and GOAT in the remnant stomach, leading to reductions in acyl-ghrelin in the circulation. In the hypothalamus, lower acyl-ghrelin signaling reduce both the GHSR usage and thus degradation, as well as the hypothalamic levels of endocannabinoids (ECs), the endogenous agonists of the orexigenic CB1R, resulting in increased levels of GHSR and in CB1R expression decreases, by 30 days post SG. In parallel, the acute loss of fat mass decreases leptin plasma levels, which in turn, and after the establishment of leptin resistance reversal (LRR),^8^ downregulate LEPRb and the levels of MC4R agonist, a-MSH. The expression of the anorexigenic MC4R did not change significantly at this time point, where the maximal weight loss is achieved post SG in humans. By 90 days post SG, a downregulation of CB1R expression in the remnant stomach results in retention of proghrelin into the A-like cells, and along with the stably low levels of GOAT sustain even lower levels of plasma acyl-ghrelin. In the hypothalamus, this effect further reduces the levels of the ECs and AgRP (endogenous MC4R antagonist); leptin levels remain stably low,^8^ so do the levels of LEPRb and the rest of the positive or negative effects on a-MSH or AgRP and ECs, respectively. Therefore, initiated by the downregulation of the acyl-ghrelin-CB1R signaling in the remnant stomach and the hypothalamus and followed by the increases in MC4R, the energy homeostasis system balances to an anorexigenic ‘set point' maintaining the lower body weight post SG (black letters illustrate results of these studies, categorized by a system where events were scored in an additive manner: one arrow was received for significant expression differences between expression in SG animals at 30 days vs starting point (0 days), 90 days or sham (SH) animals at 30 or 90 days, and two arrows for long-term significant differences, that is between expression in SG animals at 90 vs 0 days; direction of arrows indicates down or upregulation. Gray letters illustrate literature-based predicted changes in the levels of ECs, AgRP and a-MSH;→depicts activation of receptors, upregulation of receptors, or upregulation of agonist levels, and ⊣ the opposite actions).

Secretion of acyl-ghrelin by the stomach is also under the regulation of CB1R, as activation of CB1R elevates its secretion into the circulation, and blockade reduces it, whereas genetic ablation of CB1R diminishes the orexigenic effect of ghrelin.^23, 29, 31^ In turn, ghrelin prevents the downregulation of CB1R mRNA, as for example, when it counteracts the effects of postprandial cholecystokinin in nodose ganglion neurons.^32^ Therefore, we attribute the suppressed CB1R expression in the stomach (Figure 2) to lower acyl-ghrelin and to higher postprandial cholecystokinin plasma levels known to occur post SG.^7^ Moreover, ghrelin may act indirectly on the transcription of CB1R through the modulation of ECs synthesis.^23^ Thus, our data outline a temporal hierarchy in the reciprocal regulation of ghrelin secretion and CB1R expression in the stomach, whereby acute loss of stomach tissue and thus circulating ghrelin downregulates CB1R expression in the stomach, which in turn reduces the secretion of the already lower acyl-ghrelin, with the net result by 30 days, the maintenance of low acyl-ghrelin levels in the circulation (Figure 5). In the long-term, both the reduction in EC signaling and the permanent reduction of GOAT expression may lead to even lesser acyl-ghrelin in the periphery. These data highlight the role of the EC system as an effector of ghrelin actions and emphasize its importance in the success of bariatric procedures.^13^

In the hypothalamus, CB1R expression was also impaired after SG, simulating region-specific decreases of CB1R mRNA during long periods of low circulating ghrelin levels, like obesity.^11, 33^ Indeed, ghrelin promotes its orexigenic role through CB1R signaling by increasing the hypothalamic concentration of the ECs, as established in CB1R-knockout mice.^23^ Our finding that GHSR mRNA levels decreased by 90 days in the SG animals (Figure 3) further supports the hypothesis of a hierarchy, controlled by ghrelin, as its persistent low levels were needed to produce transcriptional neuroadaptations in the hypothalamus. Although this has been previously suggested by the low expression of hypothalamic GHSR in obesity,^11^ ghrelin administration has been shown to reduce or increase the expression of GHSR mRNA in primary pituitary cell cultures^17^ or in rat arcuate nucleus,^20^ respectively. These findings are not necessarily contradictory but rather suggest that this ghrelin-GHSR regulation is cell-type specific and may depend on the neuronal cellular content. In further support, we found that GHSR protein levels in the cerebellum were downregulated in parallel with its mRNA levels (Figure 4), whereas the decrease in GHSR mRNA levels in the hypothalamus paradoxically did not correlate with its protein levels. Maintenance of hypothalamic GHSR abundance persisted throughout the 3-month study period (Figure 3) and presumably reflects an expected attenuation of the degradation of GHSR due to acyl-ghrelin scarcity (Figure 5).

Low leptin levels following SG^6, 8^ are established in parallel to the progress of fat and weight loss post- SG, as the loss of fat, but not lean mass accounts for the reduction of weight.^8^ The amount of weight loss at 3 weeks post SG was sufficient to reverse leptin resistance, a significant and permanent effect.^8^ This leptin reduction may affect LEPRb expression in the hypothalamus post SG, as previously reported for weight loss produced by caloric restriction^21^ or after leptin administration that increases the expression of LEPRb in responsive but not in leptin-resistant animals.^18^ In our model, the fat mass of SG animals was significantly lower by 30 days and the loss of excess fat sustained at similar levels in the long term, thus, stably low leptin levels should be expected. The LEPRb mRNA and protein levels (Figure 3) of the SG animals were constantly lower than the sham, probably due to the fact that sham animals remained leptin resistant for longer time period. In similar studies, such changes in LEPRb mRNA expression were not recorded at least at the medio-basal part of the hypothalamus.^8^ We assessed the whole hypothalamus and thus recorded contributions from all hypothalamic areas.

Leptin resistance may also develop in the hippocampus, at least following HFD.^34^ The decreases in the hippocampal LEPRb protein levels after SG, resembling those in the hypothalamus, indicate that leptin may regulate important aspects of feeding behavior controlled by the hippocampus. Indeed, whereas hippocampus-specific ablation of LEPRb does not affect weight gain, adiposity or leptin levels,^35^ leptin signaling in the hippocampus reduces food intake and memory consolidation for the spatial location of food.^36^ In contrast to what we observed in the hypothalamus, LEPRb mRNA levels could not predict the protein levels in the hippocampus or cerebellum post SG (Figure 4). This fact demonstrates that LEPRb regulation by leptin is different in these two areas, possibly due to different affinity transportation systems of leptin,^37^ and predicts region-specific mechanisms for resistance to leptin.

In line with the observations by Stefater *et al*,^8^ we show that central nervous system mRNA expression of MC4R remained unaltered post SG. However, the protein levels of MC4R were elevated, suggesting that MC4R activity is controlled by protein availability mechanisms.^19^ This upregulation of MC4R was a late event (Figure 3) and correlated with increased LEPRb protein availability, that is with the leptin resistance reversal. Leptin resistance reversal restores the leptin positive effects on a-MSH and the negative ones on AgRP production,^12^ and, together with the low acyl-ghrelin levels, drives the net effect towards increased MC4R levels to promote the anorexigenic pathway (Figure 5). In turn, the hypothalamic neurons that express high levels of MC4R release, in response to a-MSH, the anorexigenic corticotropin-releasing hormone, which may contribute through actions on the hypothalamic-pituitary-adrenal axis^15^ in glucocorticoid-driven maintenance of lower body weight post SG.

We found a striking downregulation of MC4R in the hippocampus (Figure 4), where its actions include improvements in memory and neuroprotection.^38^ These features are suppressed during obesity, as a significant decrease of hippocampal neurons is observed in humans along with a reduction in inhibition of appetitive behavior.^39^ Bariatric surgery improves memory in humans,^40^ whereas animals subjected to SG perform better than sham animals when tested for hippocampal-dependent memory.^26^ The significant downregulation of MC4R that we detected may signify a rapid turnover of the receptor in hippocampal neurons, which is restoration of a-MSH signaling, a major mechanism in reestablishing the inhibitory memory of excess food intake, which the patients report after SG.

Summarizing, post SG and with the limitation of postoperative diet choice to only healthy low-fat diet, we observed long-term changes in the expression of appetite- and metabolism-controlling receptors, which are compatible with a reprogramming of the energy homeostasis-related pathways in the gut-brain axis to a new balance that sustained the lower body weight. We propose that: (a) both the short- and long-term changes of the acyl-ghrelin-CB1R axis primarily affected the maintenance of low EC signaling to sustain the weight loss, and (b) the changes in the leptin axis were defined by the loss of weight and had a secondary role into this new balance, as the impact of leptin signaling remained stable over time (Figure 5). Thus, peripheral intervention into the ghrelin-CB1R axis should be considered as an additional therapeutic target for human obesity in patients subjected to SG.

## Figures and Tables

**Table 1:** Post surgery gene expression analyses by reverse transcription-PCR in central nervous system (CNS) areas and the stomach

*Gene*	*Primers*	*Annealing temperature (°C)*	*CNS areas*	*Gastric tissue*
*Ghsr*	F:TCTTCGTGGTGGGCATCTCA R:AAGCAGATGGCGAAGTAGCG	51	+	
*Leprb*	F:CCAGTGCATCGCCAGGAAA R:ACAGTGAGCTGGGAATGGGC	55	+	
*Mc4r*	F:GGCTTCACATTAAGAGGATCGCT R:TTTATGGAACTCCATAGCGCC	50	+	
*Cnr1*	F:AGGGTACTTCCCACAGAAATTC R:CGTGAAGGTGCCCAGCGTGA	50	+	+
*Ghrl*	F:TCTTGAGCCCAGAGCACCAGA R:AGTTGCAGAGGAGGCAGAAGC	50		+
*Mboat4*	F:CTGGGAGGCTCCCTGTGTTCCT R:AAGTCTGCAGCACGGGCCAG	58		+
*Gapdh*	F:TCCCTCAAGATTGTCAGCAA R:AGATCCACAACGGATACATT	50	+	+

Table lists the specific primer sets and annealing temperatures used to detect the expression levels of GHSR, LEPRb, MC4R and CB1R receptors of preproghrelin (*Ghrl*) and ghrelin *O*-acyltransferase (*Mboat4*), and of the housekeeping enzyme GAPDH, in tissues indicated by the plus symbol (**+**). In all experimental groups, the hypothalamus, hippocampus, cerebral cortex, cerebellum, amygdala and olfactory bulbs were isolated according to stereotactic coordinates and individually processed.^27^ Gastric tissue was harvested from near the gastroesophageal junction of the remnant gastric tube of sleeve gastrectomized animals and from identical areas in the other study groups.

## References

[bib1] Eid GM, Brethauer S, Mattar SG, Titchner RL, Gourash W, Schauer PR. Laparoscopic sleeve gastrectomy for super obese patients: forty-eight percent excess weight loss after 6–8 years with 93% follow-up. Ann Surg 2012; 256: 262–265. 22791102 10.1097/SLA.0b013e31825fe905

[bib2] Schauer PR, Kashyap SR, Wolski K, Brethauer SA, Kirwan JP, Pothier CE et al. Bariatric surgery versus intensive medical therapy in obese patients with diabetes. N Engl J Med 2012; 366: 1567–1576. 22449319 10.1056/NEJMoa1200225 PMC3372918

[bib3] Himpens J, Dobbeleir J, Peeters G. Long-term results of laparoscopic sleeve gastrectomy for obesity. Ann Surg 2010; 252: 319–324. 20622654 10.1097/SLA.0b013e3181e90b31

[bib4] Sumithran P, Prendergast LA, Delbridge E, Purcell K, Shulkes A, Kriketos A et al. Long-term persistence of hormonal adaptations to weight loss. N Engl J Med 2011; 365: 1597–1604. 22029981 10.1056/NEJMoa1105816

[bib5] Chambers AP, Kirchner H, Wilson-Perez HE, Willency JA, Hale JE, Gaylinn BD et al. The effects of vertical sleeve gastrectomy in rodents are ghrelin independent. Gastroenterology 2013; 144: 50–2 e5. 22995675 10.1053/j.gastro.2012.09.009 PMC3752595

[bib6] Dimitriadis E, Daskalakis M, Kampa M, Peppe A, Papadakis JA, Melissas J. Alterations in gut hormones after laparoscopic sleeve gastrectomy: a prospective clinical and laboratory investigational study. Ann Surg 2013; 257: 647–654. 23108120 10.1097/SLA.0b013e31826e1846

[bib7] Peterli R, Steinert RE, Woelnerhanssen B, Peters T, Christoffel-Courtin C, Gass M et al. Metabolic and hormonal changes after laparoscopic Roux-en-Y gastric bypass and sleeve gastrectomy: a randomized, prospective trial. Obes Surg 2012; 22: 740–748. 22354457 10.1007/s11695-012-0622-3 PMC3319900

[bib8] Stefater MA, Perez-Tilve D, Chambers AP, Wilson-Perez HE, Sandoval DA, Berger J et al. Sleeve gastrectomy induces loss of weight and fat mass in obese rats, but does not affect leptin sensitivity. Gastroenterology 2010; 138: 2426–2436 36 e1-3. 20226189 10.1053/j.gastro.2010.02.059 PMC2883635

[bib9] Keidar A, Hershkop KJ, Marko L, Schweiger C, Hecht L, Bartov N et al. Roux-en-Y gastric bypass vs sleeve gastrectomy for obese patients with type 2 diabetes: a randomised trial. Diabetologia 2013; 56: 1914–1918. 23765186 10.1007/s00125-013-2965-2

[bib10] Chambers AP, Wilson-Perez HE, McGrath S, Grayson BE, Ryan KK, D'Alessio DA et al. Effect of vertical sleeve gastrectomy on food selection and satiation in rats. Am J Physiol Endocrinol Metab 2012; 303: E1076–E1084. 22932782 10.1152/ajpendo.00211.2012 PMC3469608

[bib11] Briggs DI, Enriori PJ, Lemus MB, Cowley MA, Andrews ZB. Diet-induced obesity causes ghrelin resistance in arcuate NPY/AgRP neurons. Endocrinology 2010; 151: 4745–4755. 20826561 10.1210/en.2010-0556

[bib12] Enriori PJ, Evans AE, Sinnayah P, Jobst EE, Tonelli-Lemos L, Billes SK et al. Diet-induced obesity causes severe but reversible leptin resistance in arcuate melanocortin neurons. Cell Metab 2007; 5: 181–194. 17339026 10.1016/j.cmet.2007.02.004

[bib13] Guijarro A, Osei-Hyiaman D, Harvey-White J, Kunos G, Suzuki S, Nadtochiy S et al. Sustained weight loss after Roux-en-Y gastric bypass is characterized by down regulation of endocannabinoids and mitochondrial function. Ann Surg 2008; 247: 779–790. 18438115 10.1097/SLA.0b013e318166fd5f PMC2671862

[bib14] Yang J, Brown MS, Liang G, Grishin NV, Goldstein JL. Identification of the acyltransferase that octanoylates ghrelin, an appetite-stimulating peptide hormone. Cell 2008; 132: 387–396. 18267071 10.1016/j.cell.2008.01.017

[bib15] Lu XY, Barsh GS, Akil H, Watson SJ. Interaction between alpha-melanocyte-stimulating hormone and corticotropin-releasing hormone in the regulation of feeding and hypothalamo-pituitary-adrenal responses. J Neurosci 2003; 23: 7863–7872. 12944516 10.1523/JNEUROSCI.23-21-07863.2003 PMC6740604

[bib16] Balthasar N, Dalgaard LT, Lee CE, Yu J, Funahashi H, Williams T et al. Divergence of melanocortin pathways in the control of food intake and energy expenditure. Cell 2005; 123: 493–505. 16269339 10.1016/j.cell.2005.08.035

[bib17] Luque RM, Kineman RD, Park S, Peng XD, Gracia-Navarro F, Castano JP et al. Homologous and heterologous regulation of pituitary receptors for ghrelin and growth hormone-releasing hormone. Endocrinology 2004; 145: 3182–3189. 15044357 10.1210/en.2003-1626

[bib18] Mitchell SE, Nogueiras R, Morris A, Tovar S, Grant C, Cruickshank M et al. Leptin receptor gene expression and number in the brain are regulated by leptin level and nutritional status. J Physiol 2009; 587: 3573–3585. 19491239 10.1113/jphysiol.2009.173328 PMC2742282

[bib19] Mohammad S, Baldini G, Granell S, Narducci P, Martelli AM. Constitutive traffic of melanocortin-4 receptor in Neuro2 A cells and immortalized hypothalamic neurons. J Biol Chem 2007; 282: 4963–4974. 17166828 10.1074/jbc.M608283200

[bib20] Nogueiras R, Tovar S, Mitchell SE, Rayner DV, Archer ZA, Dieguez C et al. Regulation of growth hormone secretagogue receptor gene expression in the arcuate nuclei of the rat by leptin and ghrelin. Diabetes 2004; 53: 2552–2558. 15448083 10.2337/diabetes.53.10.2552

[bib21] Wilsey J, Scarpace PJ. Caloric restriction reverses the deficits in leptin receptor protein and leptin signaling capacity associated with diet-induced obesity: role of leptin in the regulation of hypothalamic long-form leptin receptor expression. J Endocrinol 2004; 181: 297–306. 15128278 10.1677/joe.0.1810297

[bib22] Di Marzo V, Goparaju SK, Wang L, Liu J, Batkai S, Jarai Z et al. Leptin-regulated endocannabinoids are involved in maintaining food intake. Nature 2001; 410: 822–825. 11298451 10.1038/35071088

[bib23] Kola B, Farkas I, Christ-Crain M, Wittmann G, Lolli F, Amin F et al. The orexigenic effect of ghrelin is mediated through central activation of the endogenous cannabinoid system. PLoS One 2008; 3: e1797. 18335063 10.1371/journal.pone.0001797 PMC2258435

[bib24] Tam J, Cinar R, Liu J, Godlewski G, Wesley D, Jourdan T et al. Peripheral cannabinoid-1 receptor inverse agonism reduces obesity by reversing leptin resistance. Cell Metab 2012; 16: 167–179. 22841573 10.1016/j.cmet.2012.07.002 PMC3832894

[bib25] Asimaki O, Mangoura D. Cannabinoid receptor 1 induces a biphasic ERK activation via multiprotein signaling complex formation of proximal kinases PKCepsilon, Src, and Fyn in primary neurons. Neurochem Int 2011; 58: 135–144. 21074588 10.1016/j.neuint.2010.11.002

[bib26] Grayson BE, Fitzgerald MF, Hakala-Finch AP, Ferris VM, Begg DP, Tong J et al. Improvements in hippocampal-dependent memory and microglial-infiltration with calorie restriction and gastric bypass surgery but not with vertical sleeve gastrectomy. Int J Obes (Lond) 2013; 38: 349–356. 23736372 10.1038/ijo.2013.100

[bib27] Zisopoulou S, Asimaki O, Leondaritis G, Vasilaki A, Sakellaridis N, Pitsikas N et al. PKC-epsilon activation is required for recognition memory in the rat. Behav Brain Res 2013; 253: 280–289. 23911427 10.1016/j.bbr.2013.07.036

[bib28] McLaughlin D, Tsirimonaki E, Vallianatos G, Sakellaridis N, Chatzistamatiou T, Stavropoulos-Gioka C et al. Stable expression of a neuronal dopaminergic progenitor phenotype in cell lines derived from human amniotic fluid cells. J Neurosci Res 2006; 83: 1190–1200. 16555279 10.1002/jnr.20828

[bib29] Cani PD, Montoya ML, Neyrinck AM, Delzenne NM, Lambert DM. Potential modulation of plasma ghrelin and glucagon-like peptide-1 by anorexigenic cannabinoid compounds, SR141716A (rimonabant) and oleoylethanolamide. Br J Nutr 2004; 92: 757–761. 15533263 10.1079/bjn20041256

[bib30] Patrikakos P, Toutouzas KG, Gazouli M, Perrea D, Menenakos E, Papadopoulos S et al. Long-term plasma ghrelin and leptin modulation after sleeve gastrectomy in Wistar rats in comparison with gastric tissue ghrelin expression. Obes Surg 2011; 21: 1432–1437. 21611877 10.1007/s11695-011-0426-x

[bib31] Zbucki RL, Sawicki B, Hryniewicz A, Winnicka MM. Cannabinoids enhance gastric X/A-like cells activity. Folia Histochem Cytobiol 2008; 46: 219–224. 18519241 10.2478/v10042-008-0033-4

[bib32] Burdyga G, Varro A, Dimaline R, Thompson DG, Dockray GJ. Ghrelin receptors in rat and human nodose ganglia: putative role in regulating CB-1 and MCH receptor abundance. Am J Physiol Gastrointest Liver Physiol 2006; 290: G1289–G1297. 16423919 10.1152/ajpgi.00543.2005

[bib33] Timofeeva E, Baraboi ED, Poulin AM, Richard D. Palatable high-energy diet decreases the expression of cannabinoid type 1 receptor messenger RNA in specific brain regions in the rat. J Neuroendocrinol 2009; 21: 982–992. 19807847 10.1111/j.1365-2826.2009.01921.x

[bib34] Valladolid-Acebes I, Merino B, Principato A, Fole A, Barbas C, Lorenzo MP et al. High-fat diets induce changes in hippocampal glutamate metabolism and neurotransmission. Am J Physiol Endocrinol Metab 2012; 302: E396–E402. 22114023 10.1152/ajpendo.00343.2011

[bib35] Guo M, Lu Y, Garza JC, Li Y, Chua SC, Zhang W et al. Forebrain glutamatergic neurons mediate leptin action on depression-like behaviors and synaptic depression. Transl Psychiatry 2012; 2: e83. 22408745 10.1038/tp.2012.9 PMC3298113

[bib36] Kanoski SE, Hayes MR, Greenwald HS, Fortin SM, Gianessi CA, Gilbert JR et al. Hippocampal leptin signaling reduces food intake and modulates food-related memory processing. Neuropsychopharmacology 2011; 36: 1859–1870. 21544068 10.1038/npp.2011.70 PMC3154104

[bib37] Zlokovic BV, Jovanovic S, Miao W, Samara S, Verma S, Farrell CL. Differential regulation of leptin transport by the choroid plexus and blood-brain barrier and high affinity transport systems for entry into hypothalamus and across the blood-cerebrospinal fluid barrier. Endocrinology 2000; 141: 1434–1441. 10746647 10.1210/endo.141.4.7435

[bib38] Giuliani D, Mioni C, Altavilla D, Leone S, Bazzani C, Minutoli L et al. Both early and delayed treatment with melanocortin 4 receptor-stimulating melanocortins produces neuroprotection in cerebral ischemia. Endocrinology 2006; 147: 1126–1135. 16254026 10.1210/en.2005-0692

[bib39] Davidson TL, Chan K, Jarrard LE, Kanoski SE, Clegg DJ, Benoit SC. Contributions of the hippocampus and medial prefrontal cortex to energy and body weight regulation. Hippocampus 2009; 19: 235–252. 18831000 10.1002/hipo.20499 PMC2649976

[bib40] Alosco ML, Spitznagel MB, Strain G, Devlin M, Cohen R, Paul R et al. Improved memory function two years after bariatric surgery. Obesity (Silver Spring) 2013; 22: 32–38. 23625587 10.1002/oby.20494 PMC4054602

